# A Deep Learning and XGBoost-Based Method for Predicting Protein-Protein Interaction Sites

**DOI:** 10.3389/fgene.2021.752732

**Published:** 2021-10-26

**Authors:** Pan Wang, Guiyang Zhang, Zu-Guo Yu, Guohua Huang

**Affiliations:** ^1^ School of Electrical Engineering, Shaoyang University, Shaoyang, China; ^2^ Key Laboratory of Intelligent Computing and Information Processing of Ministry of Education and Hunan Key Laboratory for Computation and Simulation in Science and Engineering, Xiangtan University, Xiangtan, China

**Keywords:** protein-protein interaction, deep learning, machine learning, extreme gradient boosting, protein functions

## Abstract

Knowledge about protein-protein interactions is beneficial in understanding cellular mechanisms. Protein-protein interactions are usually determined according to their protein-protein interaction sites. Due to the limitations of current techniques, it is still a challenging task to detect protein-protein interaction sites. In this article, we presented a method based on deep learning and XGBoost (called DeepPPISP-XGB) for predicting protein-protein interaction sites. The deep learning model served as a feature extractor to remove redundant information from protein sequences. The Extreme Gradient Boosting algorithm was used to construct a classifier for predicting protein-protein interaction sites. The DeepPPISP-XGB achieved the following results: area under the receiver operating characteristic curve of 0.681, a recall of 0.624, and area under the precision-recall curve of 0.339, being competitive with the state-of-the-art methods. We also validated the positive role of global features in predicting protein-protein interaction sites.

## Introduction

Proteins are one of the most important components of the cell, and also are the principal undertaker of the activities of life. The functions of proteins are manifested mainly by interacting with various molecules such as DNA/RNA, proteins, or other ligands (Dias and Kolaczkowski, 2017). The protein-protein interaction (PPI) plays a key role in the cellular process such as signal transduction, transport, and metabolism (Li et al., 2019) and also is involved in the pathogenesis of diseases such as Alzheimer’s cervical cancer, bacterial infection, and prion diseases (Cohen and Prusiner, 1998; Selkoe, 1998; Loregian et al., 2002). Therefore, knowledge of PPI is critical for understanding the molecular mechanisms hidden in the phenomenon of life (Das and Chakrabarti, 2021). Many experimentally verified or computationally predicted PPIs have been hosted for scientific research in public databases such as the Human Protein Reference Database (Keshava Prasad et al., 2009), STRING (Von Mering et al., 2005), the database of interacting proteins (Salwinski et al., 2004), and the protein interaction database (Kerrien et al., 2007). The protein-protein interaction site (PPIS) is defined as surface residues where proteins interact with each other (Aumentado-Armstrong et al., 2015). The identification of PPIS is the premise for determining PPI (Wang et al., 2019). The knowledge about PPIS holds vast potential to infer cell regulatory mechanisms, locate drug targets, identify structures and functions of protein complexes (Deng et al., 2009; Orii and Ganapathiraju, 2012), and uncover disease pathogenesis (Kuzmanov and Emili, 2013). Drug discovery and development are also closely associated with PPIS (Sperandio, 2012; Petta et al., 2016). Therefore, identifying PPIS is of great importance in the field of molecule biology.

It is not only costly but also time-consuming and labor-intensive to identify PPIS by experimental methods such as alanine scanning mutagenesis and crystallographic complex determination (Aumentado-Armstrong et al., 2015; Krï¿ ½ger and Gohlke, 2010; Bradshaw et al., 2011). Since Jones and Thornton pioneered a computational method for predicting and analyzing PPIS in 1997 (Jones and Thornton, 1997; Jones and Thornton, 1997), more than thirty other computational methods have been developed (Zhou and Shan, 2001; Fernandez-Recio et al., 2004; Neuvirth et al., 2004; Bradford and Westhead, 2005; Chen and Zhou, 2005; Chung et al., 2006; Liang et al., 2006; Patel et al., 2006; Li et al., 2007; Ofran and Rost, 2007; Porollo and Meller, 2007; Qin and Zhou, 2007; Tjong et al., 2007; Chen and Jeong, 2009; Dosztányi et al., 2009; Du et al., 2009; Engelen et al., 2009; Šikić et al., 2009; Fiorucci and Zacharias, 2010; Murakami and Mizuguchi, 2010; Shoemaker et al., 2010; Segura et al., 2011; Xue et al., 2011; Zhang et al., 2011; Chen et al., 2012; Jordan et al., 2012; La and Kihara, 2012; Li et al., 2012; Qiu and Wang, 2012; Zellner et al., 2012; Bendell et al., 2014; de Moraes et al., 2014; Singh et al., 2014; Wang et al., 2014; Aumentado-Armstrong et al., 2015; Bagchi, 2015; Dayal et al., 2015; Maheshwari and Brylinski, 2015; Dick and Green, 2016; Jia et al., 2016; Kuo and Li, 2016; Wei et al., 2016; Hou et al., 2017; Zhao et al., 2017; Guo et al., 2018; Northey et al., 2018; Wang et al., 2019; Wang et al., 2019; Zhang and Kurgan, 2019; Zhang and Kurgan, 2019; Deng et al., 2020; Li, 2020; Zeng et al., 2020; Zhu et al., 2020; Wang et al., 2021; Wang et al., 2021). Due to their efficiency, computational methods are becoming essentially complementary to experimental methods. Most computational methods for identifying PPIS are based on machine learning algorithms where the prediction performance depends heavily on learning algorithms and feature extractions. The learning algorithms used for PPIS prediction generally include conditional random fields (Li et al., 2007), support vector machines (Bradford and Westhead, 2005), random forest (Chen and Jeong, 2009), XGBoost (Deng et al., 2020), logistic regression (Zhang and Kurgan, 2019), Bayes method (Murakami and Mizuguchi, 2010), and artificial neural networks (Singh et al., 2014). These learning algorithms are not suitable for enough large number of training samples. Recently, deep learning algorithms have been developed that have achieved significant superiority over traditional learning algorithms, especially in many difficult cases such as image classification (Krizhevsky et al., 2012; He et al., 2016) and protein structure prediction (Callaway, 2020). Features used for PPIS prediction generally include evolutionary information (Caffrey et al., 2004; Carl et al., 2008; Choi et al., 2009), secondary structure (Guharoy and Chakrabarti, 2007; Ofran and Rost, 2007; Li et al., 2012) and physicochemical, biophysical and statistical features such as accessible surface area (de Vries and Bonvin, 2008; Hou et al., 2017) and backbone flexibility (Bendell et al., 2014). According to its source, features are divided into sequence-based, structure-based, and hybrid features, which are a combination of sequence and structure features (Zeng et al., 2020). The sequence-based feature is cheaper to calculate but does not contain any information from structures that might be responsible for protein functions. The structures of most proteins are not available, while structural information generally obtained by computational prediction contain noise, which sometimes heavily effected subsequent discrimination. Information from neighboring residues of interaction sites is important to determine protein-protein interaction sites. In addition, there exists binding signals far from interaction sites. Zeng et al. (2020) demonstrated that inclusion of global features increased the performance of predicting protein-protein interaction sites. Both the local and the global features were obtained by non-linear degeneration. That is to say, during the transformation from proteins to features, information is lost. In addition, the local and the global features also contained noise. The deep learning-based encoder answers these issues above. Inspired by this, we used the DeepPPISP proposed by Zeng et al. (2020) to refine features of protein-protein interaction sites, Extreme Gradient Boosting (XGBoost) to learn a classifier for unknown PPIS prediction.

## Datasets

For a fair comparison with other state-of-the-art methods, we used the same three datasets as in the literature (Zeng et al., 2020). These datasets are named respectively Dset_186, Dset_72 (Murakami and Mizuguchi, 2010), and Dset_164 (Singh et al., 2014). The procedure of collecting them is briefly described as follows. All the data originated from the PDB database (Berman et al., 2000). Dset_186, Dset_72 and Dset_164 consisted of 186, 72, and 164 non-repetitive protein sequences with the resolution less than 3.0 Å, respectively. In each dataset, sequence homology between any two sequences was less than 25%. Three datasets were integrated, containing in total 422 protein sequences. Two proteins had no definition of secondary structure of proteins (DSSP) file without which their features cannot be computed. Thus these two protein sequences were removed by Zeng et al. (2020). Finally, the remaining 420 protein sequences were used.

Protein-protein interaction binding sites are determined by the absolute solvent accessibility of amino acids. If the absolute solvent accessibility was less than 1 Å^2^, the amino acid was considered to be a binding site, and otherwise it was a non-interaction site. There were 5,517, 6,096, and 1,923 binding sites, as well as 30,702, 27,585, and 16,217 non-interaction sites in the Dset_186, Dset_164, and Dset_72 datasets respectively. 83.3% of the protein sequences were randomly selected as the training set and 16.7% of the protein sequences as the testing set. The training set was further divided into two parts: 90% of the training set was used for training and 10% was used for verification. Finally, 300 protein sequences were used for training (containing 65,869 amino acid residues), 50 protein sequences for verification (containing 7,319 amino acid residues), and 70 protein sequences for independent testing (containing 11,791 amino acid residues) (Zeng et al., 2020).

## Methods

**FIGURE 1:**
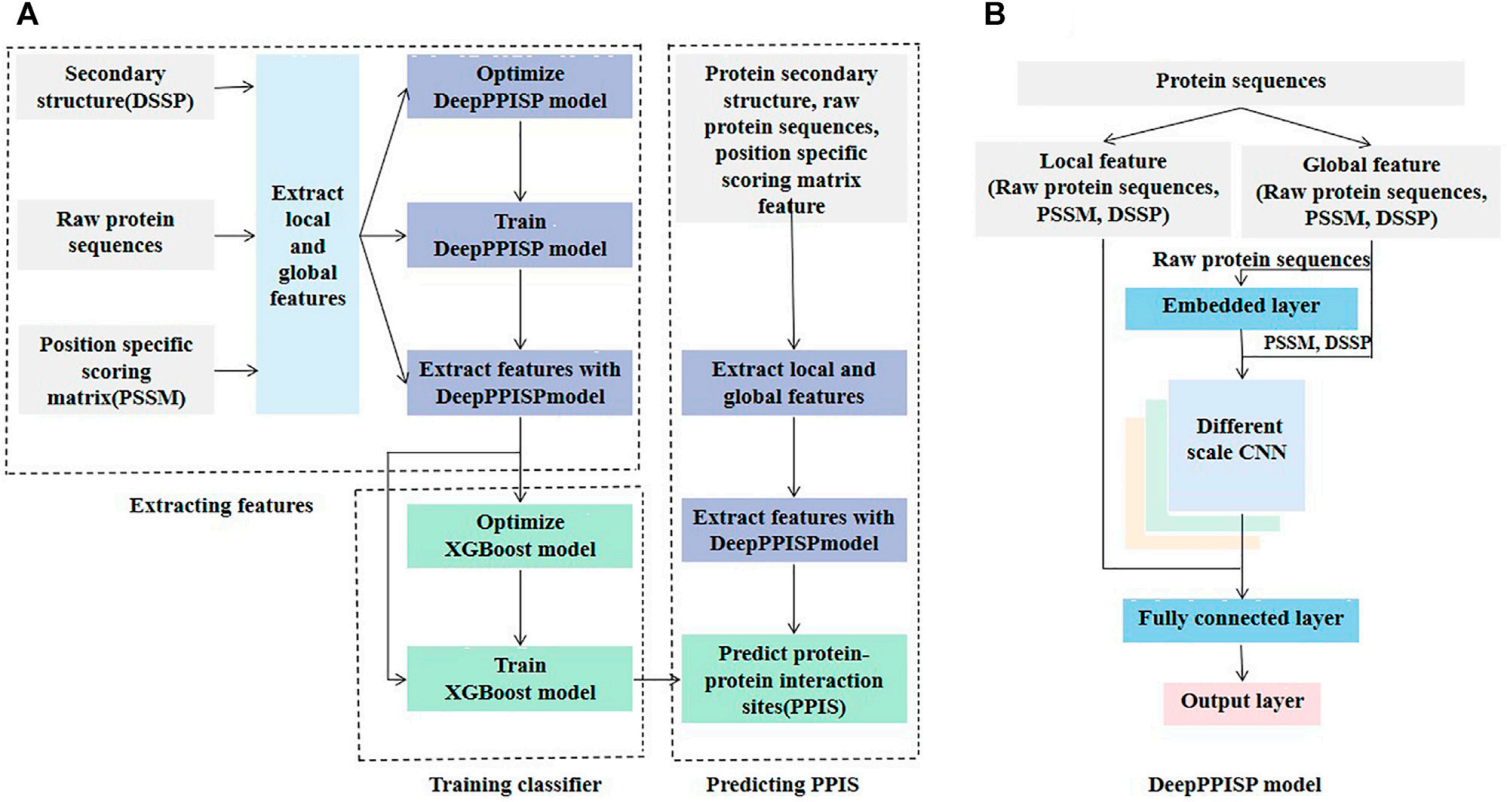
The architecture of DeepPPISP-XGB model. **(A)** Illustration of the DeepPPISP-XGB workflow, which consists of three modules: extracting feature, training classifier, predicting PPIS. **(B)** The architecture of DeepPPISP model, which contain embedding layer, different scale convolutions, fully connected layers and output layer.

The proposed method called DeepPPISP-XGB consisted of three main steps: extracting features, training a classifier, and predicting PPIS (Figure 1A). The DeepPPISP was a deep learning model proposed by Zeng et al. (Zeng et al., 2020) for PPIS (Figure 1B). Here, we used it as an encoder of amino acid sequences, because the deep learning algorithms have a powerful ability to represent objects. We trained the DeepPPISP model with the training set. The input of the first fully connected layer in the trained DeepPPISP was used as a representation of the input. The XGBoost classifier was trained by the preprocessing features of the encoder. For unknown protein sequences which have secondary structure, raw protein sequence, and position-specific scoring matrix feature, the trained DeepPPISP extracted preprocessing features firstly and then the trained XGBoost classifier predicted PPIS.

### DeepPPISP

As shown in Figure 1B, the DeepPPISP proposed by Zeng et al. (Zeng et al., 2020) for PPIS prediction had three types of input: position-specific scoring matrix (PSSM), secondary structure, and raw protein sequences. The PSSM is an excellent feature extractor for protein sequences and thus have widely been applied to problems in the field of computational biology, such as predicting protein post-translational modification (Huang et al., 2013; Huang et al., 2014; Dehzangi et al., 2017), membrane type (Wang et al., 2019), protein-RNA binding site (Liu et al., 2021), and structure (Guo et al., 2021). The quality of PSSM features is closely associated with the underlying multiple sequence alignments. Although there are many multiple sequence alignment algorithms including HIMMER (Eddy, 2011; Wheeler and Eddy, 2013) (Johnson et al., 2010) and Hhbilits (Remmert et al., 2012), PSI-BLAST (Altschul et al., 1997) is still a popular multiple sequence alignment and homology search algorithm. Here, PSI-BLAST was used to search NCBI’s non-redundant (NR) sequence database with three iterations and an E-value threshold of 0.001.

Many protein-protein interfaces are related to secondary structures (Taechalertpaisarn et al., 2019). Information about protein secondary structure is helpful to predict PPIS. The DSSP program (Touw et al., 2015) was used to generate nine state secondary structures: *α*-helix, 3_10_- helix, π-helix, β-bridge, β-strand, β-turn, bend, loop or irregular, and no secondary structure. Therefore, each amino acid residue corresponded to a 9-dimensional vector. The primary protein sequence is valuable information and thus is essential to predict protein properties. One-hot encoding was used to encode the protein sequences. There are 20 kinds of common amino acids in the protein sequences, so each amino acid residue corresponds to a 20-dimensional 0/1 vector. The protein-protein interaction is closely associated with neighboring residues of interaction sites. The local feature of interaction sites contributes to the identification of PPIS. The sliding window method was used to collect the neighboring residues of the interaction sites. The size of the sliding window was seven. For example, if the interaction site was at position i, residues at position i-3, i-2, i-1, i, i+1, i+2, and i+3 were separated. Because each residue corresponds to a 20-dimensional PSSM feature, a 9-dimensional secondary structure feature, and a 20-dimensional one-hot feature vector, a window of seven amino acid residues was encoded into a 343-dimensional vector which was called the local feature.

Protein-protein interaction is not only linked to the local information of interacting sites, but also to global information. Zeng et al. (2020) demonstrated that the inclusion of global information improved the performance of predicting PPIS. A 500-residue peptide was used to represent the global feature of PPIS. If the number of amino acid residues in the protein sequence was less than 500, it was padded with a 0. Each peptide corresponds to a 500*49-dimensional vector called a global feature.

The local and the global features were fed into the DeepPPISP (Zeng et al., 2020). The DeepPPISP was made up of one embedding layer, three different scale convolutions, two fully connected layers, and an output layer (Figure 1B). For more detail, readers can refer to the reference (Zeng et al., 2020).

Both the local features or global features would contain a certain degree of noise. The dimension is large, especially for global features. The DeepPPISP was used to extract a more informative representation. The DeepPPISP was trained on the training data in a supervised manner. The local and global features were fed into the trained DeepPPISP, and the input to the first fully connected layer was the abstract representation of the raw features. Compared with the raw features, the abstract representation was of low dimension and had low noise.

### XGBoost Algorithm

The XGBoost proposed by Chen and Guestrin, 2016 belongs to Gradient Boosting Decision Tree (GBDT) (Ke et al., 2017), and both are tree boosting algorithms. Compared with traditional tree boosting, the XGBoost used a theoretically justified weighted quantile sketch for approximate learning, a novel sparsity aware algorithm for handling sparse data, and an effective cache-aware block structure for out-of-core tree learning (Chen and Guestrin, 2016). In addition, the XGBoost performed faster as it exploited parallel and distributed computing. The XGBoost has such a significant superiority that it has widely been used in many areas including machine learning and data mining challenges.

The XGBoost is an addition model. At each iteration, the XGBoost learns a new tree that fits the residual between the predicted result of the previous trees and the true values of the training samples.

Assume that $D=\{\left(x_{i},y_{i}\right)|\left|D\right|=n,x_{i}\in R^{m},y_{i}\in R\}$ denotes a training set, where m and n represented the numbers of features and samples, respectively. At the t-th iteration, the aim of the XGBoost is to learn a function $f_{t}$ so that

$${\hat{y}}_{i}^{t}={\hat{y}}_{i}^{t-1}+f_{t}\left(x_{i}\right)$$ (1)

where ${\hat{y}}_{i}^{t-1}$ is the fitting value of the previous t−1 trees for the i-th sample. To search for $f_{t}$ , the loss function with the regularization was used as the objective function:

$$\mathrm{obj}=\;\overset{n}{\underset{i=1}{\sum }}l\left(y_{i},{\hat{y}}_{i}^{t}\right)+\overset{n}{\underset{i=1}{\sum }}\Omega \left(f_{t}(x_{i})\right)=\overset{n}{\underset{i=1}{\sum }}l\left(y_{i},{\hat{y}}_{i}^{t-1}+f_{t}\left(x_{i}\right)\right)+\Omega \left(f_{t}\right)+\mathrm{constant},$$ (2)

where l was the loss function which was generally defined as

$$l\left(y_{i},{\hat{y}}_{i}^{t}\right)={\left(y_{i}-{\hat{y}}_{i}^{t}\right)}^{2}.$$ (3)

$\overset{t}{\underset{i=1}{\sum }}\text{Ω}\left(f_{i}\right)$ denotes the regularization. The loss function l was approximated by the second-order Taylor series, namely

$$l\left(y_{i},{\hat{y}}_{i}^{t-1}+f_{t}\left(x_{i}\right)\right)\approx l\left(y_{i},{\hat{y}}_{i}^{t-1}\right)+g_{i}f_{t}\left(x_{i}\right)+\frac{1}{2}h_{i}f_{t}^{2}\left(x_{i}\right),$$ (4)

where $g_{i}=\frac{\partial l(\left(y_{i},{\hat{y}}_{i}^{t-1}\right)}{\partial {\hat{y}}_{i}^{t-1}}$ and $h_{i}=\frac{\partial^{2}l(\left(y_{i},{\hat{y}}_{i}^{t-1}\right)}{\partial {\hat{y}}_{i}^{t-1}\partial {\hat{y}}_{i}^{t-1}}$ were the first- and the second-order gradients of the loss function with respect to ${\hat{y}}_{i}^{t-1}$ respectively. $\text{Ω}\left(f_{t}\right)$ was defined by

$$\Omega \left(f_{t}\right)=\mathrm{\gamma T}+\frac{1}{2}\overset{T}{\underset{j=1}{\sum }}\omega_{j}^{2},$$ (5)

where T was the number of leaf nodes and $\omega_{j}$ was the weight of the j-th leaf node. The objective function was equivalently rewritten as

$$\mathrm{obj}=\overset{n}{\underset{i=1}{\sum }}\left[g_{i}f_{t}\left(x_{i}\right)+\frac{1}{2}h_{i}f_{t}^{2}\left(x_{i}\right)\right]+\mathrm{\gamma T}+\frac{1}{2}\lambda \overset{T}{\underset{j=1}{\sum }}\omega_{j}^{2}.$$ (6)

The set of instances of the leaf node j was defined by

$$I_{j}=\left\{x_{i}\text{|}q\left(x_{i}\right)=j\right\}.$$ (7)

The objective function was further represented as

$$\mathrm{obj}=\overset{T}{\underset{j=1}{\sum }}\left(\sum_{i\in I_{j}}g_{i}\right)\omega_{j}+\frac{1}{2}\overset{T}{\underset{j=1}{\sum }}\left(\sum_{i\in I_{j}}h_{i}+\lambda \right)\omega_{j}^{2}+\mathrm{\gamma T}$$ (8)

Given a fixed tree q(x) , the optimal value of each leaf node was calculated by

$$\omega_{j}^{*}=\frac{\sum_{i\in I_{j}}g_{i}}{\sum_{i\in I_{j}}h_{i}+\lambda },$$ (9)

and the optimal value of the whole tree was calculated by

$$\mathrm{ob}j^{*}=-\frac{1}{2}\overset{T}{\underset{j=1}{\sum }}\frac{{\left(\sum_{i\in I_{j}}g_{i}\right)}^{2}}{\sum_{i\in I_{j}}h_{i}+\lambda }+\mathrm{\gamma T}.$$ (10)

It was expensive and impossible to exhaust all the possible trees for the training data. In practice, the greedy algorithm was used, which started from one node and iteratively split the node. Assume that before the node was split, the objective function of the tree was

$$\mathrm{ob}j_{1}=-\frac{1}{2}\overset{T-1}{\underset{j=1}{\sum }}\frac{{\left(\sum_{i\in I_{j}}g_{i}\right)}^{2}}{\sum_{i\in I_{j}}h_{i}+\lambda }+\mathrm{\gamma T}-\frac{1}{2}\frac{{\left(\sum_{i\in I_{k}}g_{i}\right)}^{2}}{\sum_{i\in I_{k}}h_{i}+\lambda }.$$ (11)

After the node k was split into the left tree $\;I_{L}$ and the right tree $I_{R}$ , the objective function was

$$\mathrm{ob}j_{2}=-\frac{1}{2}\overset{T-1}{\underset{j=1}{\sum }}\frac{{\left(\sum_{i\in I_{j}}g_{i}\right)}^{2}}{\sum_{i\in I_{j}}h_{i}+\lambda }+\gamma \left(T+1\right)\;-\frac{1}{2}\frac{{\left(\sum_{i\in I_{L}}g_{i}\right)}^{2}}{\sum_{i\in I_{L}}h_{i}+\lambda }—\frac{1}{2}\frac{{\left(\sum_{i\in I_{R}}g_{i}\right)}^{2}}{\sum_{i\in I_{R}}h_{i}+\lambda }.$$ (12)

The gain of node splitting was calculated by

$$\mathrm{gain}=\mathrm{ob}j_{1}-\mathrm{ob}j_{2}=\frac{1}{2}\frac{{\left(\sum_{i\in I_{L}}g_{i}\right)}^{2}}{\sum_{i\in I_{L}}h_{i}+\lambda }+\frac{1}{2}\frac{{\left(\sum_{i\in I_{R}}g_{i}\right)}^{2}}{\sum_{i\in I_{R}}h_{i}+\lambda }-\frac{1}{2}\frac{{\left(\sum_{i\in I_{k}}g_{i}\right)}^{2}}{\sum_{i\in I_{k}}h_{i}+\lambda }-\gamma .$$ (13)

The gain was used to assess the split candidates.

## Evaluation Metrics

In the area of machine learning, the frequently used evaluation metrics include accuracy (ACC), Recall, Precision, F1-score (F1), and Matthews correlation coefficient (MCC) which are respectively calculated by the following formulas:

$$\mathrm{ACC}=\frac{\mathrm{TP}+\mathrm{TN}}{\mathrm{TP}+\mathrm{FP}+\mathrm{TN}+\mathrm{FN}}$$ (14)

$$\mathrm{Recall}=\frac{\mathrm{TP}}{\mathrm{TP}+\mathrm{FN}}$$ (15)

$$\mathrm{Precision}=\frac{\mathrm{TP}}{\mathrm{TP}+\mathrm{FP}}$$ (16)

$$F1=2\times \frac{\mathrm{Sensitivity}\times \mathrm{Precision}}{\mathrm{Sensitivity}+\mathrm{Precision}}$$ (17)

$$\mathrm{MCC}=\frac{\mathrm{TP}\times \mathrm{TN}-\mathrm{FP}\times \mathrm{FN}}{\sqrt{\left(\mathrm{TP}+\mathrm{FP}\right)\times \left(\mathrm{TP}+\mathrm{FN}\right)\times \left(\mathrm{TN}+\mathrm{FP}\right)\times \left(\mathrm{TN}+\mathrm{FN}\right)}}$$ (18)

where TP and TN denote respectively the numbers of the true positive and the true negative samples, and FP and FN denote the numbers of the false positive and false negative samples. The F1-score ranges from 0 to 1. F1-score values close to 1 indicated the best prediction. The MCC represents the correlation coefficient between the actual classification and the predicted classification. The range of MCC values is −1 to 1, where 1 meant perfect prediction, and −1 indicated the worst prediction. The area under the receiver operating characteristic curve (AUROC) and area under the precision-recall curve (AUPRC) were also used to evaluate the performances.

## Experiments

### Visualization of Preprocessing Features

**FIGURE 2:**
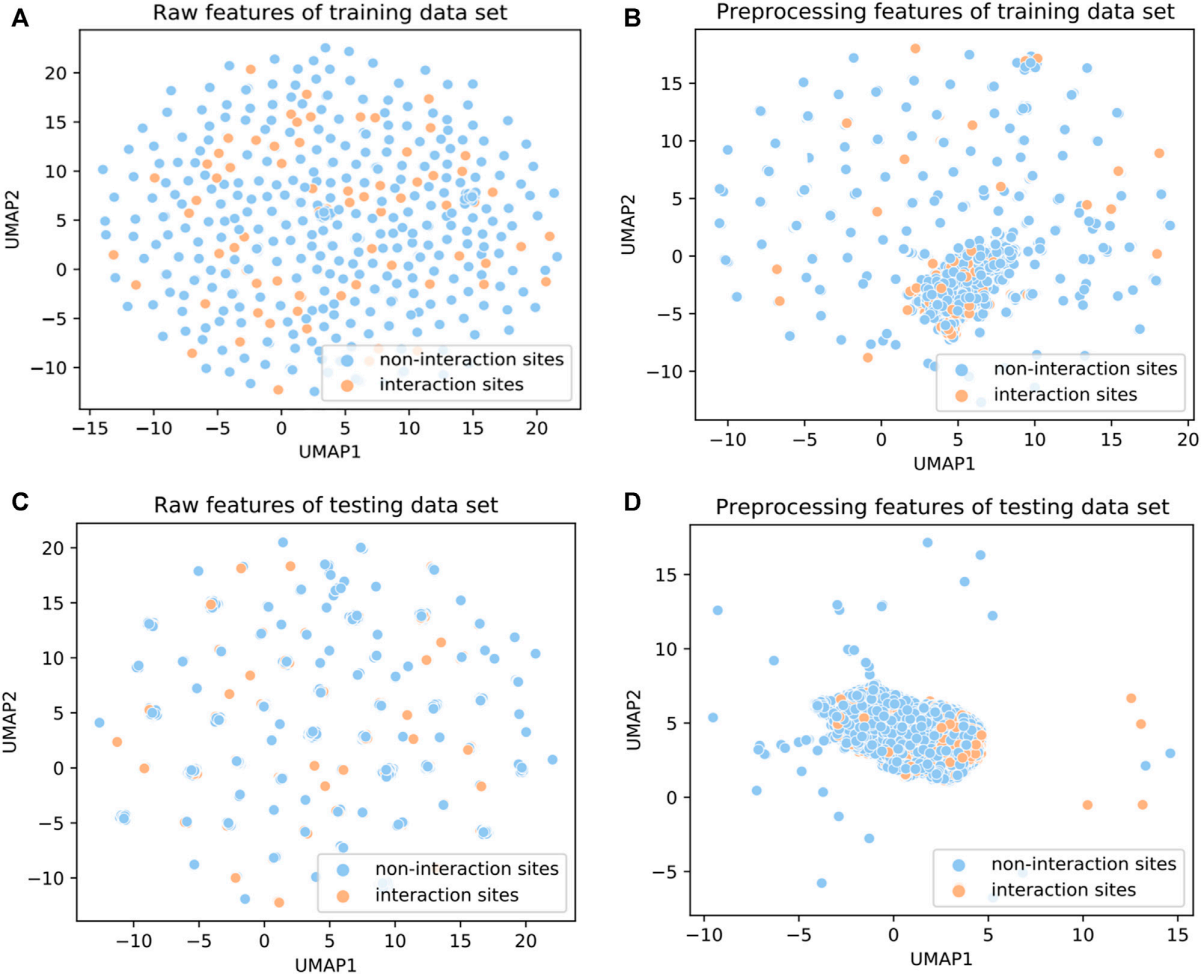
UMAP diagrams of **(A)** raw features of the training set, **(B)** preprocessing features of the training set, **(C)** raw features of the testing set, and **(D)** preprocessing feature of the testing set.

**FIGURE 3:**
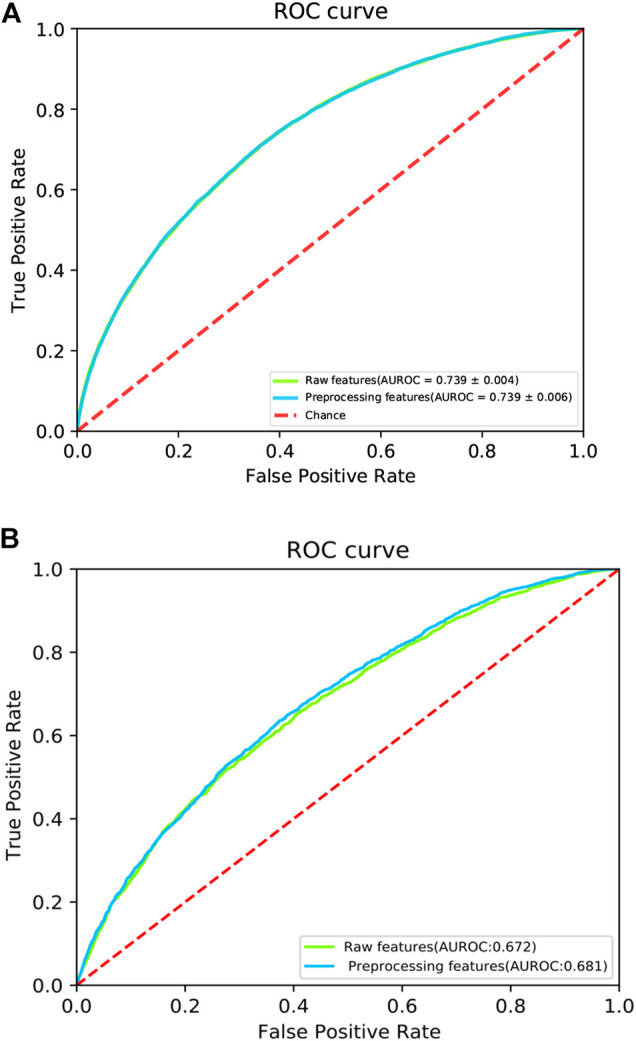
The ROC curves of **(A)** 5-fold cross validation and **(B)** independent test. The red dotted line is a control line on which AUROC = 0.5.

To investigate the ability of the features to discriminate protein-protein interaction sites from non-interaction sites, we used the Uniform Manifold Approximation and Projection (UMAP) (McInnes et al., 2020) to depict the first two principal components. The UMAP is a powerful tool for dimension reduction and visualization. As shown in Figure 2, the features processed by the DeepPPISP demonstrated a tighter cluster than the raw features, indicating that features generated by the DeepPPISP were more discriminative. To further evaluate the performance of the preprocessed features, we performed 5-fold cross-validation and independent tests. Figure 3A showed the ROC curves of the 5-fold cross-validation over both the preprocessing features and raw features, while Figure 3B depicted the ROC curves of the independent tests. The performance of preprocessed features is equivalent to or better than those of raw features. It must be pointed out that the user-defined parameters were identical in the XGBoost classifiers. Comparison with other methods

Due to its versatile roles in the cellular process, the identification of protein-protein interaction sites is increasingly becoming a hot topic and is also a challenging task. Over the past decades, more than 10 methods have been proposed to predict protein-protein interaction sites (Patel et al., 2006; Du et al., 2009; Murakami and Mizuguchi, 2010; Wang et al., 2014; Zhang and Kurgan, 2019; Northey et al., 2018; Zeng et al., 2020; Chen et al., 2012; Šikić et al., 2009; Fiorucci and Zacharias, 2010; Dosztányi et al., 2009; La and Kihara, 2012; Bradford and Westhead, 2005; Chen and Jeong, 2009; Chung et al., 2006; Fernandez-Recio et al., 2004; Shoemaker et al., 2010; Ofran and Rost, 2007; Qin and Zhou, 2007; Liang et al., 2006; Li et al., 2007; Zhou and Shan, 2001; Neuvirth et al., 2004; Porollo and Meller, 2007; Segura et al., 2011; Qiu and Wang, 2012; Wei et al., 2016; Zhu et al., 2020; Guo et al., 2018; Kuo and Li, 2016; Wang et al., 2021; Maheshwari and Brylinski, 2015; Li, 2020; Dick and Green, 2016; Wang et al., 2019; Zhao et al., 2017; Jia et al., 2016; Deng et al., 2020; Singh et al., 2014; Hou et al., 2017; Li et al., 2012; Wang et al., 2019; Bagchi, 2015 #412; Zhang and Kurgan, 2019). We compared the proposed method with six other state-of-the-art methods. These six competing methods were DeepPPISP (Zeng et al., 2020), SCRIBER (Zhang et al., 2019), IntPred (Northey et al., 2018), RF_PPI (Hou et al., 2017), SPRINGS (Singh et al., 2014), PSIVER (Murakami and Mizuguchi, 2010), ISIS (Ofran and Rost, 2007), and SPPIDER (Porollo and Meller, 2007). PSIVER was a Naïve Bayes-based classifier that used features from PSSM and accessibility, while SPPIDER combined fingerprints with information from the sequences and structures for PPIS predictio. Both SPRINGS and ISIS were neural network-based methods. The former used evolutionary information, averaged cumulative hydropathy, and predicted relative solvent accessibility, while the latter used structural features and evolutionary information. RF_PPI was a random forest-based classifier for PPIS prediction, while the DeepPPISP was a deep learning-based classifier. The performances of these seven methods over the independent test were listed in Table 1.

**TABLE 1:** Comparison with other state-of-the-art methods.

Method	ACC	Precision	Recall	F1	AUROC	AUPRC	MCC
SPPIDER^a^	0.622	0.209	0.459	0.287	—	0.23	0.089
ISIS^a^	**0.694**	0.211	0.362	0.267	—	0.24	0.097
PSIVER^a^	0.653	0.253	0.468	0.328	—	0.25	0.138
SPRINGS^a^	0.631	0.248	*0.598*	0.35	—	0.28	0.181
RF.PPI^a^	0.598	0.173	0.512	0.258	—	0.21	0.118
IntPred^a^	*0.672*	0.247	0.508	0.332	—	—	0.165
SCRIBER^a^	0.616	0.274	0.569	0.37	0.635	0.307	0.159
DeepPPISP^a^	0.655	**0.303**	0.577	*0.397*	*0.671*	*0.32*	*0.206*
DeepPPISP-XGB	0.633	*0.296*	**0.624**	**0.402**	**0.681**	**0.339**	**0.209**

a Results reported by DeepPPISP (Zeng et al., 2020).

The highest results are highlighted in bold and the second-highest results are marked in italics. Values that were not reported by the corresponding source are indicated by “—”.

The DeepPPISP-XGB method achieved the highest value in terms of Recall, F1-score, AUROC, AUPRC, and MCC, and it reached the second-highest performance in terms of Precision. Although ISIS got the best ACC, its performance in other respects was lower than those of DeepPPISP-XGB. The DeepPPISP-XGB method improved the Recall by 4.7%, 5.5%, 11.6%, 11.2%, 2.6%, 15.6%, 26.2%, and 16.5%, in comparison with DeepPPISP, SCRIBER, IntPred, RF.PPI, SPRINGS, PSIVER, ISIS, and SPPIDER, respectively. The DeepPPISP-XGB method increased F1-score and MCC by 0.5% and 0.3%, and the AUROC by 1%, in comparison with DeepPPISP.

**FIGURE 4:**
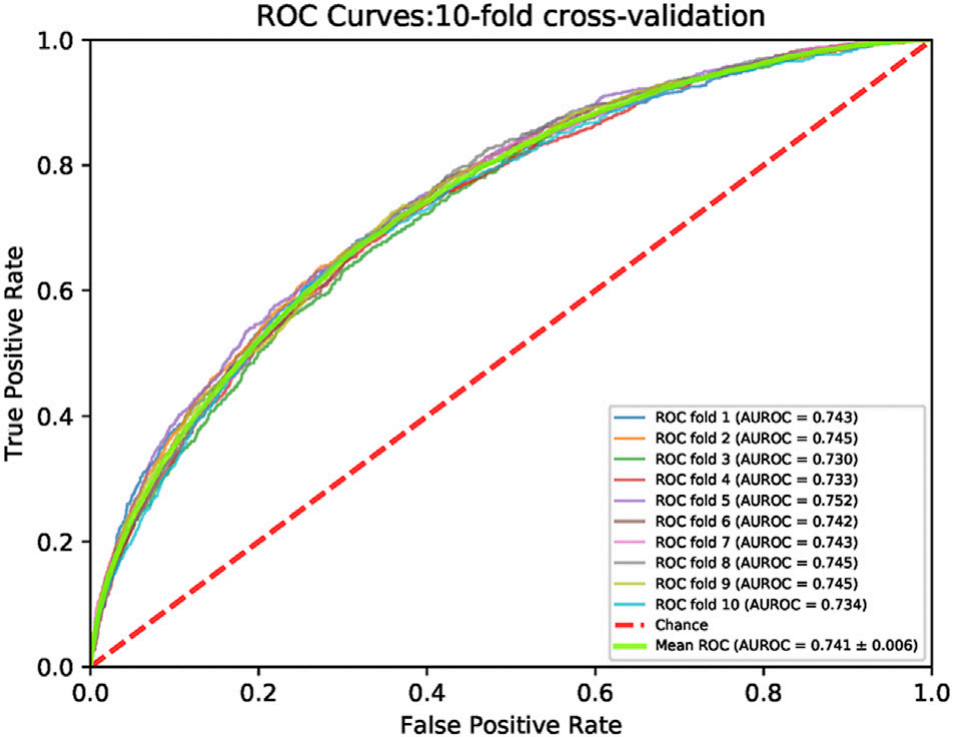
The ROC curves of 10-fold cross validation on the train set. The minimum AUROC value cross validation is 0.730 at the first fold. The maximum value of the cross validation is 0.752 at the ten-th fold. The green line represents the ROC curve of the cross validation mean. The mean value of AUROC is 0.741. The red dotted line is a control line on which AUROC = 0.5.

K-fold cross-validation is a common method in regression or classification questions. In the k-fold cross-validation, the training set was split into k parts. One part was tested and other k−1 parts were trained. The procedure was performed k times. We carried out 10-fold cross-validations, and the principle was shown (Supplementary Figure S1). Figure 4 showed ROC curves for the 10-fold cross-validations. The mean and the standard deviation of the AUROCs were 0.741 and 0.006, respectively. Supplementary Table S1 lists the ACC, Precision, Recall, F1-score, AUROC, AUPRC, and MCC for each cross-validation.

**FIGURE 5:**
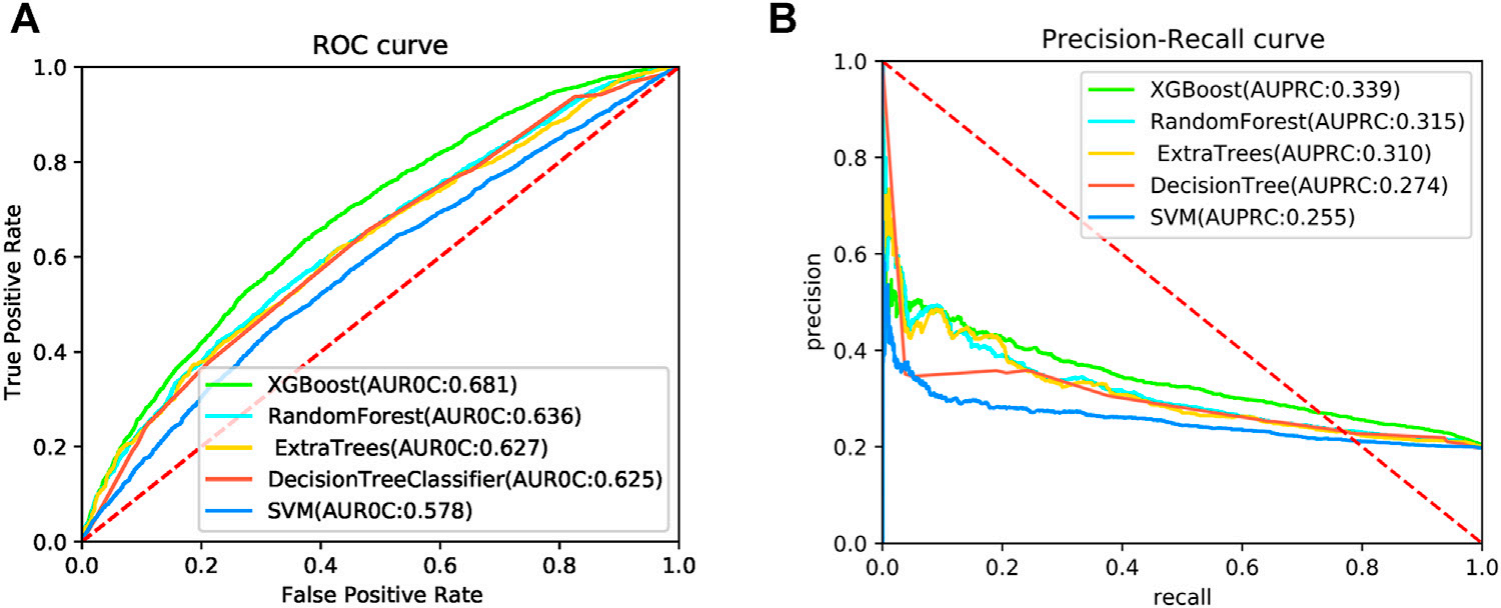
The ROC curves **(A)** and precision-recall curves **(B)** for 5 algorithms on the independent test.

To further evaluate the predictive performance of the DeepPPISP-XGB method, four machine learning algorithms were used for PPIS prediction. Decision tree (Safavian and Landgrebe, 1991) is a widely utilized classification algorithm, which is made up of the root node, internal nodes, and leaf node. Random forest (RF) (Breiman, 2001) is an ensemble learning algorithm. It consists of many weak classifiers which determine the sample category. Extremely randomized tree (ERT) (Geurts et al., 2006) is similar to RF but the decision tree of ERT is randomly divided. Support vector machine (SVM) is a statistical algorithm proposed by Boser et al. (Boser et al., 1992). These classifiers were implemented in the Scikit-Learn package (v0.24.2) which has been widely utilized in computational biology. The ROC curves and the precision-recall curves are shown in Figure 5. The XGBoost classifier obtained an AUROC value of 0.681 and an AUPRC value of 0.339 on the independent test, significantly better than four classifiers.

### The Effects of the Global Features

**FIGURE 6:**
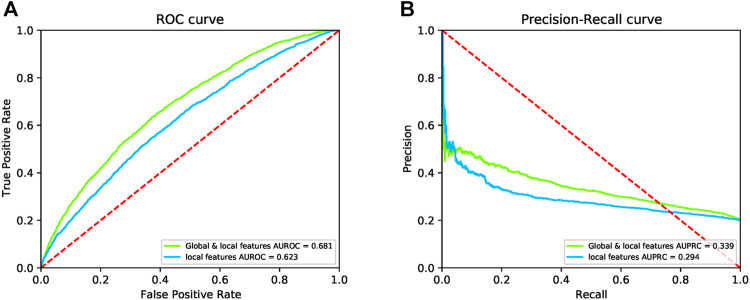
The ROC curves **(A)** and the precision-recall curves **(B)** for both local and global & local features on the independent test.

After removing global features, we trained DeepPPISP-XGB. The user-defined parameters of the DeepPPISP-XGB were the same as the previous. Table 2 shows the performance of predicting PPIS by using local features alone. The ROC and the precision-recall curves were displayed in Figure 6. The experimental results showed that the inclusion of the global features was beneficial to improve PPIS prediction, which was in agreement with the findings of Zeng et al. (2020).

**TABLE 2:** Predictive performance when using local features and using combined local and global features with the DeepPPISP-XGB model.

Features	ACC	Precision	Recall	F1	MCC
Local features	0.654	0.276	0.461	0.345	0.138
Global & local features	0.633	0.296	0.624	0.402	0.209

## Conclusion

We presented a PPIS prediction algorithm based on the DeepPPISP and the XGBoost. The DeepPPISP served as a feature extractor to remove redundant information of the protein sequences. The XGBoost was used to construct a classifier for predicting PPIS. The DeepPPISP-XGB achieved competitive performances with other state-of-the-art methods.

## Source Code

Source code is available at: https://github.com/fatancy2580/DeepPPISPXGB-master.

## Data Availability

The original contributions presented in the study are included in the article/Supplementary Material, further inquiries can be directed to the corresponding author.
