# Supplementary figures and images for: A Deep Learning and XGBoost-Based Method for Predicting Protein-Protein Interaction Sites
Source: Front Genet. 2021 Oct 26;12:752732. doi: 10.3389/fgene.2021.752732 (PMC8576272; doi:10.3389/fgene.2021.752732)

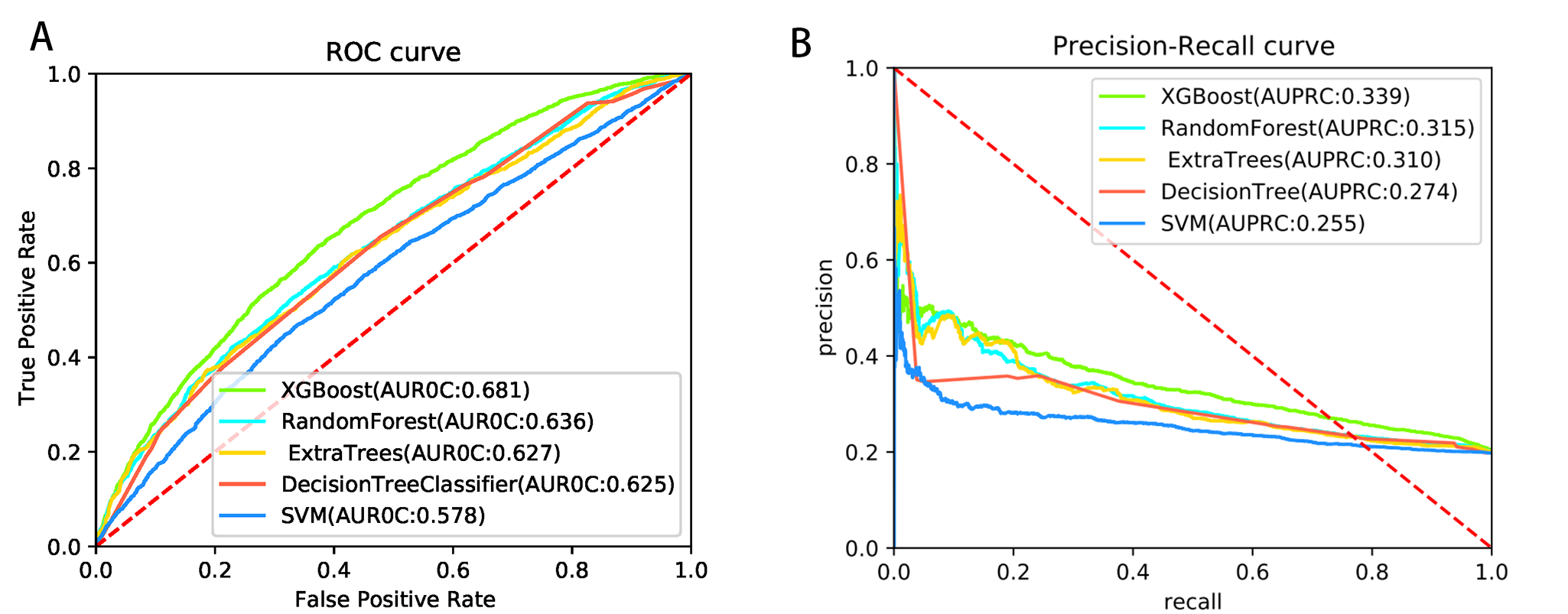

Supplement: Supplementary file 1 [file Figure5.TIF]

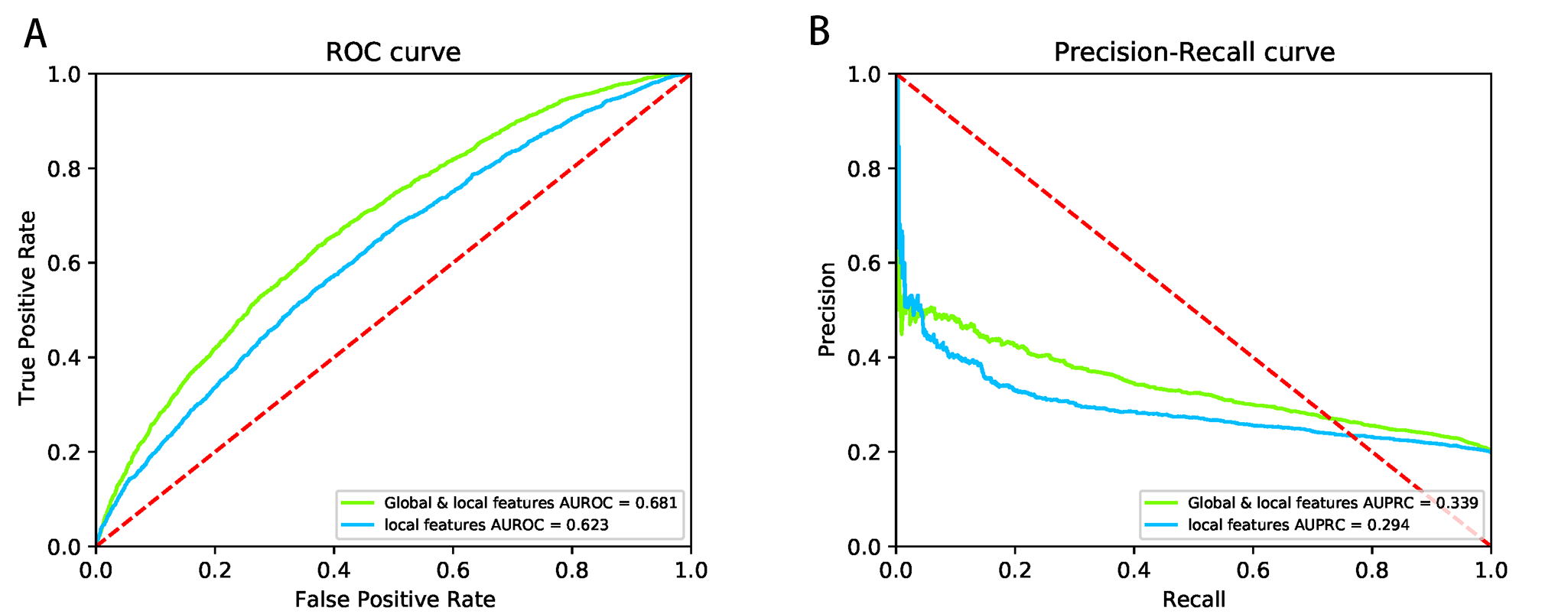

Supplement: Supplementary file 2 [file Figure6.TIF]

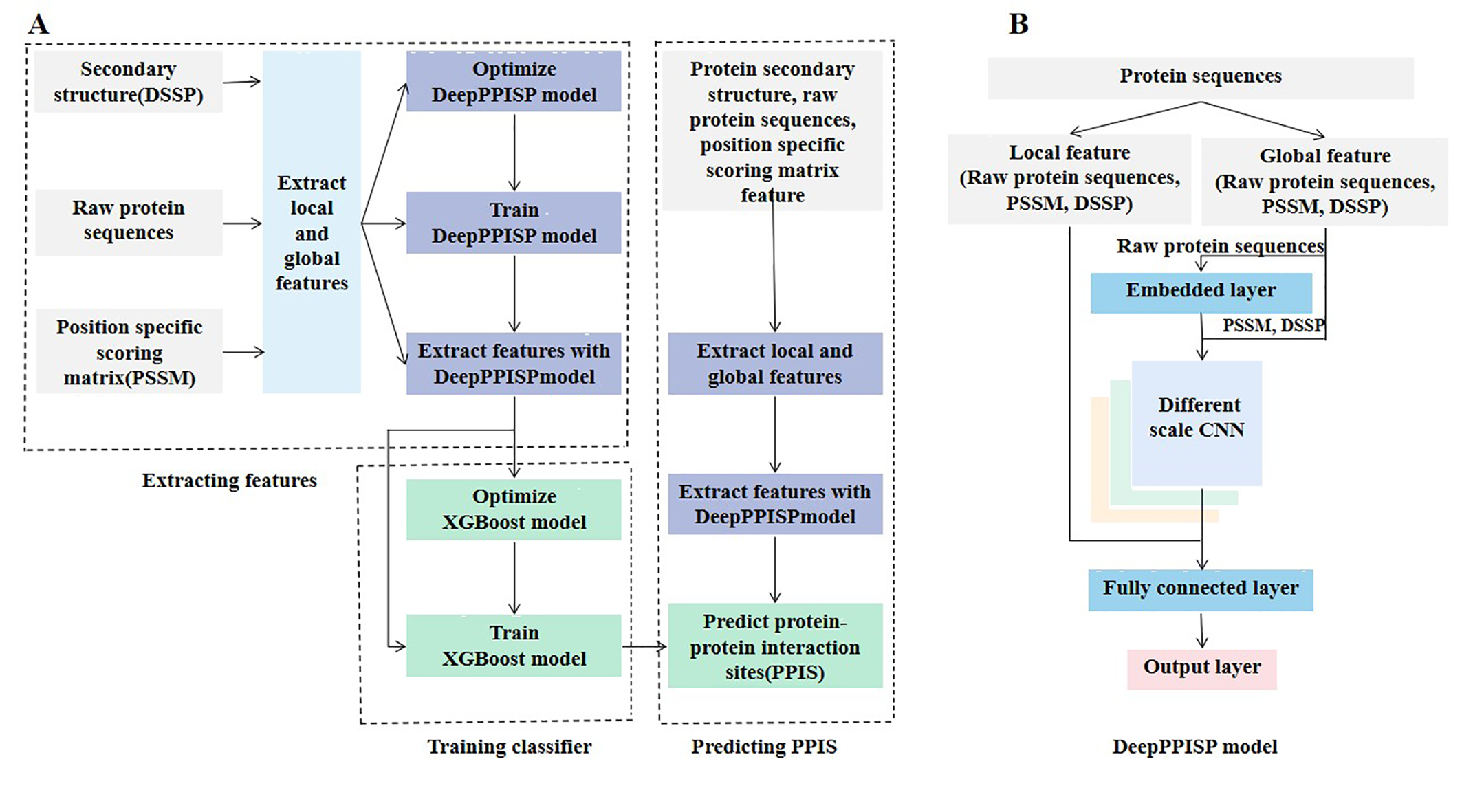

Supplement: Supplementary file 3 [file Figure1.TIF]

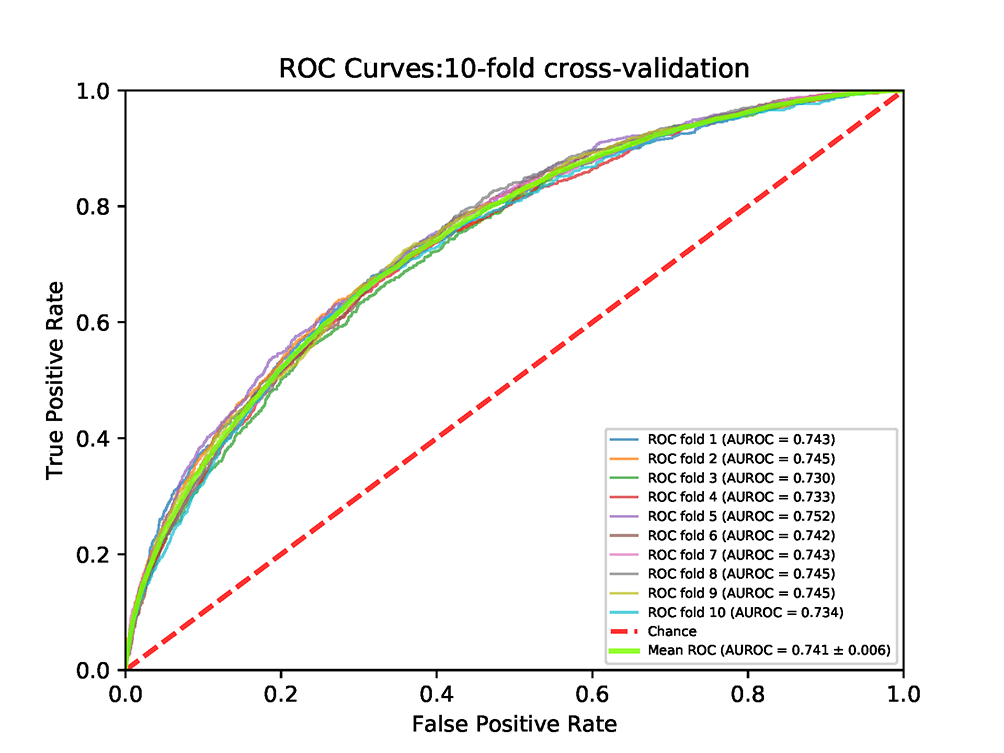

Supplement: Supplementary file 4 [file Figure4.TIF]

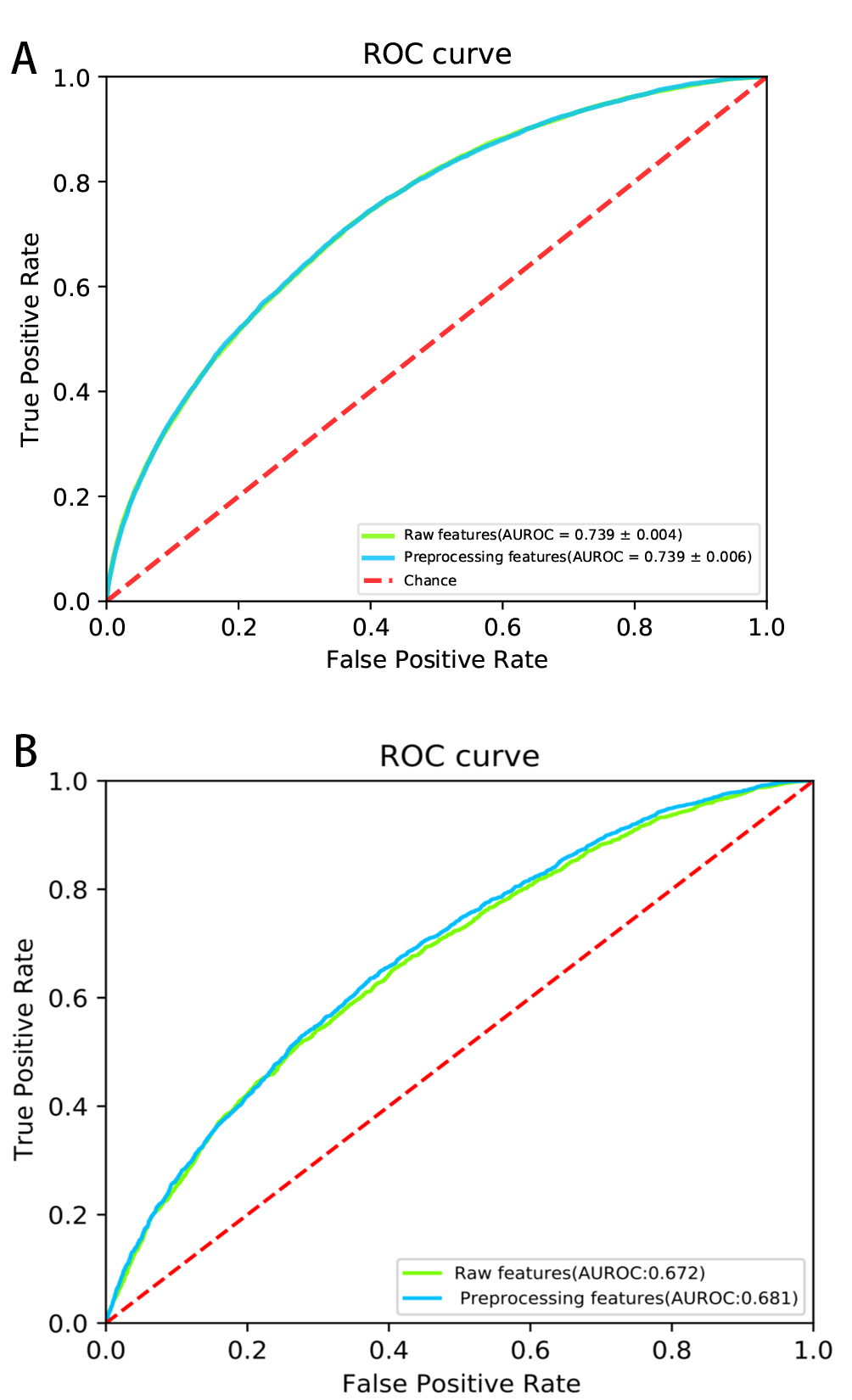

Supplement: Supplementary file 5 [file Figure3.TIF]

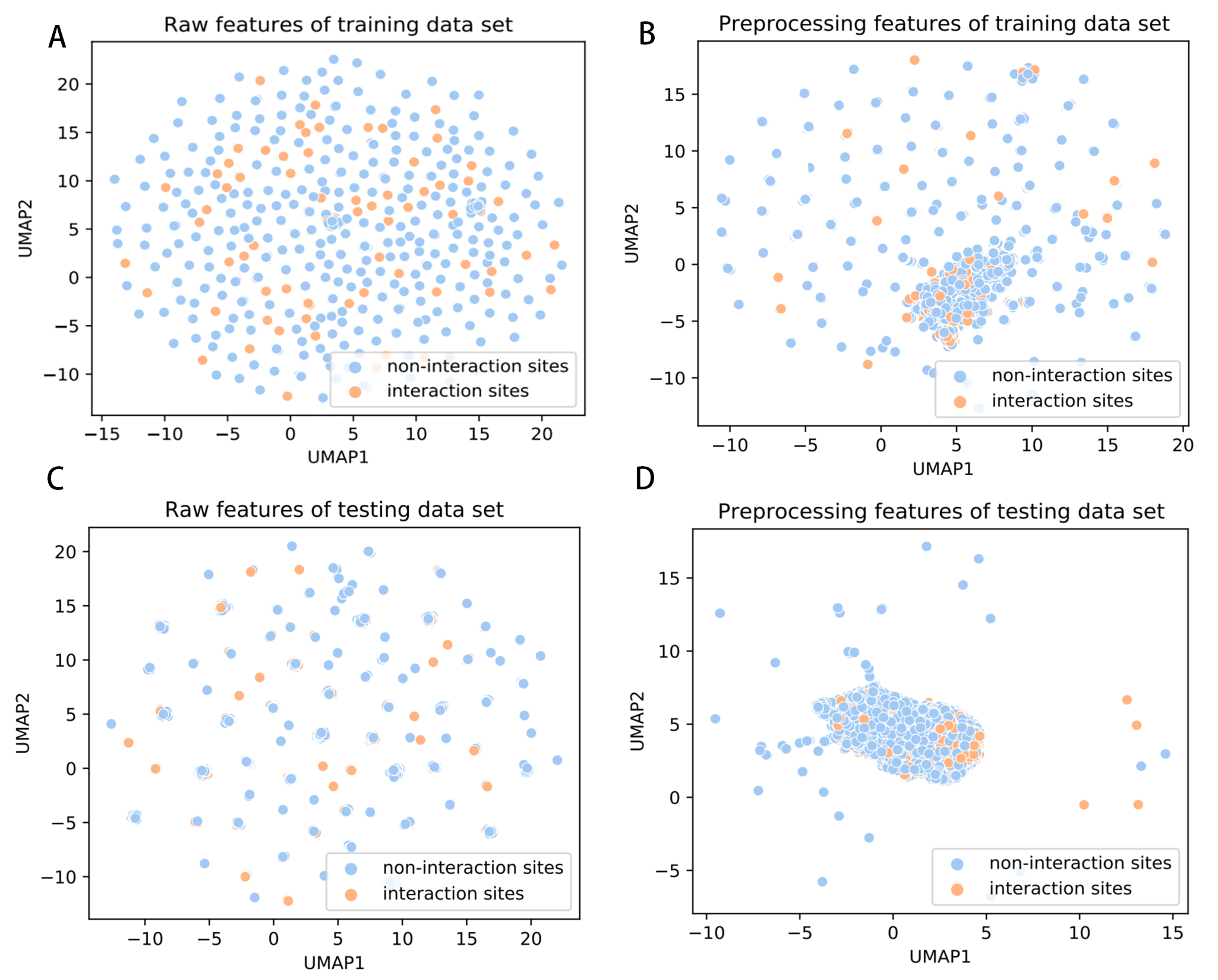

Supplement: Supplementary file 7 [file Figure2.TIF]
