# Supplementary material for: A Deep Learning and XGBoost-Based Method for Predicting Protein-Protein Interaction Sites
Source: Front Genet. 2021 Oct 26;12:752732. doi: 10.3389/fgene.2021.752732 (PMC8576272; doi:10.3389/fgene.2021.752732)
Supplement: Supplementary file 6 [file DataSheet1.PDF]

Supplementary Material

1 Supplementary Figures and Tables

1.1 Supplementary Figures

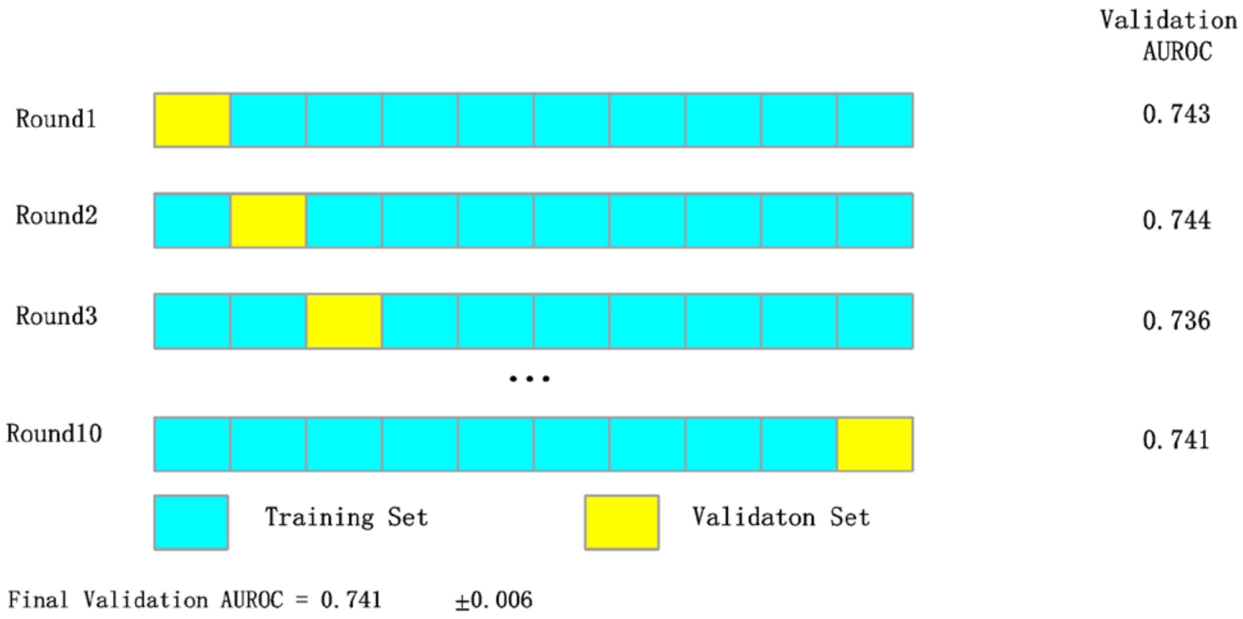

Supplementary Figure 1. The principle of cross validation

1.2 Supplementary Tables

Supplementary Tables 1. The predicting performance of 10-fold cross validation. Mean fold represents the Mean of the predicting performance of 10-fold cross validation

fold	ACC	Precision	Recall	F1	AUROC	AUPRC	MCC
fold1	0.734	0.301	0.578	0.396	0.743	0.371	0.268
fold2	0.744	0.310	0.572	0.402	0.745	0.380	0.277
fold3	0.736	0.297	0.552	0.386	0.730	0.347	0.256
fold4	0.730	0.294	0.569	0.388	0.733	0.347	0.258
fold5	0.742	0.310	0.584	0.405	0.752	0.371	0.281
fold6	0.737	0.298	0.553	0.387	0.742	0.346	0.257
fold7	0.741	0.303	0.559	0.393	0.743	0.373	0.265
fold8	0.742	0.304	0.554	0.392	0.745	0.357	0.264
fold9	0.734	0.295	0.551	0.384	0.745	0.372	0.252
fold10	0.741	0.303	0.560	0.393	0.734	0.350	0.265
Mean fold	0.738	0.302	0.563	0.393	0.741	0.361	0.264
